# Stay at Home! Governance Quality and Effectiveness of Lockdown

**DOI:** 10.1007/s11205-021-02742-3

**Published:** 2021-06-28

**Authors:** Vincenzo Alfano, Salvatore Ercolano

**Affiliations:** 1 Department of Structures for Engineering and Architectures, University of Napoli Federico II, Napoli, Italy; 2 Center for Economic Studies - CESifo, Munich, Germany; 3 Department of Mathematics, Computer Science, and Economics, University of Basilicata, Potenza, Italy

**Keywords:** COVID-19, Lockdown, Non-pharmaceutical intervention, Effects of governance, I18, H1, P48

## Abstract

In order to control the spread of the COVID-19 pandemic, during the first wave of the pandemic numerous countries decided to adopt lockdown policies. It had been a considerable time since such measures were last introduced, and the first time that they were implemented on such a global scale in a contemporary, information intensive society. The effectiveness of such measures may depend on how citizens perceive the capacity of government to set up and implement sound policies. Indeed, lockdown and confinement policies in general are binding measures that people are not used to, and which raise serious concerns among the population. For this reason governance quality could affect the perception of the benefits related to the government’s choice to impose lockdown, making citizens more inclined to accept it and restrict their movements. In the present paper we empirically investigate the relation between the efficacy of lockdown and governance quality (measured through World Governance Indicators). Our results suggest that countries with higher levels of government effectiveness, rule of law and regulatory quality reach better results in adopting lockdown measures.

**Supplementary Information:**

The online version contains supplementary material available at 10.1007/s11205-021-02742-3.

## Introduction

Following the outbreak of Coronavirus Infectious Disease (COVID-19) in China in December 2019, by April 2020 the contagion had spread to 213 countries. The COVID-19 pandemic appears to be different from the last notable epidemic (SARS), both in term of contagiousness and lethality rate. According to the World Health Organization (WHO), by 12 May 2020 there were more than 4 million confirmed cases across the world, and approximately 280,000 deaths.

The risks related to this virus stress countries’ healthcare systems, threatening their capacity to efficaciously treat the infected. Governments’ responses to such criticism have been heterogeneous across countries (Piguillem & Shi, 2020). Numerous countries, in order to ease the strain on health services by slowing down the outbreak (Hamzelou, 2020), have adopted lockdown policies alongside policies aimed at directly strengthening the hospital system. The former policy, possibly the oldest non-pharmaceutical intervention applied by governments (Alfano & Sgobbi, 2021), aims to reduce the probability of people contracting the virus. Lockdown measures are considered very important because of the difficulty in identifying the chain of transmission (Munster et al., 2020), which is caused by the possibility of asymptomatic carriers infecting other people without realizing it (Rothe et al., 2020). According to Shao (2020), health policies and lockdown policies are deeply interconnected: indeed, “adding a large number of hospital beds, along with city lockdown, improved cure rates and reduced mortality rates.”

It is the first time in a number of years that lockdowns have been implemented, and possibly the first time that such a policy has been put in place in most countries around the world, as well as the first time such measures have been introduced on a widespread basis in a deeply interconnected and information intensive society. Despite the fact that lockdowns have been implemented relatively recently, several contributions have already emerged in the literature, aimed at assessing how this type of policy works, along with its efficacy in terms of containing contagion. The first studies, which analyse the Chinese case, have highlighted the importance and centrality of such measures to reduce the probability of contagion. Lau et al. (2020) state that “a significantly decreased growth rate and increased doubling time of cases was observed, which is most likely due to Chinese lockdown measures”, and suggests a more stringent confinement of people in high-risk areas in order to control the spread of COVID-19. Shao (2020), by means of a SAIRD model, adds some observations regarding the efficacy of lockdown in China, highlighting the complementarity between policies aimed at increasing the number of hospital beds and lockdown.

Looking at the Indian case, Sardar et al. (2020) have less univocal findings. Using a SIR model that incorporates lockdown, the authors conclude that positive effects of the lockdown are observed only in some provinces. A recent study from the Istituto Superiore di Sanità and the Bruno Kessler Foundation of Trento, analysing the reproduction trend of the virus, “underlines the importance of non-pharmaceutical control measures” (Riccardo et al., 2020).

From a cross-country perspective, Alfano and Ercolano (2020a) suggest the efficacy of lockdown measures in slowing down the number of new cases. Nevertheless, the effectiveness of such measures may depend on how citizens perceive the capacity of government to set up and implement sound policies. Indeed, lockdown and confinement policies in general are binding measures that people are unaccustomed to. Moreover, according to Lau et al. (2020), lockdown could raise serious concerns among the population, as these kinds of policies recall grave historical moments. For this reason, governance quality may affect the perception of the benefits related to the government’s choice to impose lockdown, and citizens may be more inclined to accept it, and accordingly restrict their movements. If this mechanism is at work, governance quality could be able to affect the efficacy of such measures, driven by greater compliance related to the recognition of proper institutions, as observed by certain studies (Marien and Hooghe, 2011; Torgler et al., 2007). In this perspective, it may be useful to recall the central issue pointed out by Perry et al. (2014), in which the authors state: “What constitutes good governance? We generally subscribe to a simple principle: good governance is that which contributes to the good of society” (p. 27).

Following this rationale, the present contribution tries to understand whether the efficacy of measures such as lockdowns depends on the relationship between citizens and “the traditions and institutions by which authority in a country is exercised”, according to the definition of governance proposed by Kaufmann et al. (2010).

More specifically, it might be supposed that different individuals’ perception of governance quality may affect the effectiveness of lockdown. To investigate this point, the present study attempts to verify the following research hypotheses:

- **H1**: Countries characterized by greater confidence in the quality of policy formulation and the government’s commitment to such policies can implement more effective lockdowns;
- **H2**: The country’s capacity to ensure the rules of society, representing an incentive to effectively respect the lockdown, foster the effectiveness of lockdown.
- **H3**—Countries where the government is perceived as having the capacity to implement sound policies can implement more effective lockdowns;

The rest of the paper is as follows: after this brief introduction, Sect. 2 provides the main coordinates regarding governance quality, trust, compliance and policy effectiveness. Section 3 presents data and methodology, while Sect. 4 reports the main results, and Sect. 5 presents robustness tests. In Sect. 6 we present the conclusions of the study.

## Background

According to Koppell and Auer (2012), governance is a word that contains several conceptions regarding the means to deliver public ends; however, it also represents a broadened conception of the ways in which the public interest is addressed. Various scholars have studied the effect of governance and institutional quality on a number of different outcomes. Torgler and Schneider (2009), supposing that citizens perceive institutions in terms of representation of their interests, found support regarding the capacity of societal institutions to limit the extent of a shadow economy.

Governance quality has been associated by several scholars with individual compliance. In their seminal work, Torgler et al. (2007) explored the determinants of tax morale, which is a factor that is positively correlated with tax compliance. According to the authors, governance quality—and more specifically voice and accountability, rule of law, political stability and absence of violence, regulatory quality and control of corruption—shows a positive and strong effect on tax morale. Their findings seem to be confirmed in the case of developing countries too. In a study of Nigeria, Alabede and Zainal Affrin (2011) discovered that public governance quality shows a significant positive relationship with tax compliance. However, a recent study also suggested that, in the case of developing countries especially, the relationship between governance quality and tax compliance is mediated by socioeconomic conditions (Umar et al., 2019).

Following this rationale, Marien and Hooghe (2011) suggest that better institutions tend to incentivize individuals’ compliance with the law by means of political trust, which positively reflects the effectiveness and legitimacy of government action.

According to Letki (2006), the institutional dimension, “both in the form of individuals’ perceptions as well as the quality of governance”, matters in fostering civic morality. The positive effect of proper institutions on individual trust and social capital can also be diffused over time across generations (Ljunge, 2014). It is worth noting that the positive effect of institutional quality on interpersonal trust may also be a driver fostering policy effectiveness; as pointed out by Zac and Knack (2001), Knack and Zak (2003) trust is an endogenous factor aimed at minimizing transaction costs in the event of asymmetric and costly information. According to Ramírez de la Cruz et al. (2020), when transaction costs are present, collaborative efforts are threatened by the costs of creating, monitoring and enforcing the supply of public goods and services. The same authors detect in the transaction costs associated with institutional arrangements between different levels of government one of the possible factors able to affect intergovernmental relations based on trust.

In this rationale it is possible to assume that the quality of governance perceived by individuals, which positively affects the level of trust, is also able to influence policy effectiveness. This is particularly important when looking at those policies, like lockdowns, where individuals are asked to change their habits considerably, and accept strong limitations on their freedom. Bargain and Aminjonov (2020), examining the COVID case, suggest that trust affect individuals’ compliance. Their recent study, using data on individual mobility, found that trust in policymakers is positively correlated with a reduction in mobility, affecting the effectiveness of such stringent policies. However, this relation may also depend on other factors. Chan et al. (2020), who highlight the COVID-19 crisis as a “global, and (mostly) simultaneous test of the behavioural implications arising from this confidence” (p. 1), suggest that trust in policymakers may also be mediated by confidence in the healthcare system, which is able to signal citizens’ awareness of the country’s capacity to handle the pandemic. Their findings suggest that societies characterized by trust in the government and low levels of confidence in the healthcare system show a better behavioural response to the stay-at-home requirements. Nevertheless, it is worth noting that some scholars have suggested that “external” factors may also affect policy response to the COVID-19 pandemic. In particular, according to Bickley et al. (2020), countries with higher governance capacity must also face their exposition to globalization, which may slow down the adoption of stringent policies. The authors conclude that if this is the case, such governments may be pushed to adopt “learn from others” behaviour. It is evident that the COVID-19 crisis, which is a “global, and (mostly) simultaneous test of the behavioural implications arising from this confidence” (Chan et al., 2020, p. 1), is pushing social scientists to a deeper theoretical and empirical investigation of the possible mechanisms that, by means of trust and compliance, are able to connect institutional factors, namely governance quality, with policy effectiveness.

## Data and Methodology

To measure the efficacy of lockdown in a cross-country perspective, we relied on the models available in the literature. Thus, following Alfano and Ercolano (2020a, 2020b) we chose to utilize a panel dataset, with daily data from countries all over the world used as the basic statistical unit of observation. In formal terms, we estimate the following equation:

1 $$ \Delta i_{{ct}}  = \alpha  + \beta _{1} i_{{ct - 1}}  + \beta _{2} DLD_{{ct}}  + \varepsilon $$

where $\Delta i$ are new COVID-19 cases at time *t* with respect to *t-1* in country *c*. The daily increase in the spread of infection in country *c* is modelled as a function of the infections measured the previous day ($i_{{t - 1}}$). The equation also includes DLD, a dummy signalling whether or not on day *t* country *c* was under lockdown. Alternatively, DLD may signal whether a lockdown was in place for *x* days (this will be explained more fully later), in order to control for the efficiency of the policy over time.

To estimate the equation, we need: the daily number of COVID-19 cases, daily data on lockdown measures, and quality of government proxies. The former are gathered from the ‘Novel Coronavirus Cases’ dataset compiled by the Johns Hopkins University Center for Systems Science and Engineering (Dong et al., 2020), in its latest version available at the time of writing, on 24 March 2021. It offers a daily estimation of COVID-19 cases; we focused on the first wave, from 22 January to 31 May 2020. From this source we computed *New cases pc*, the operationalization of $\Delta i$, as the first difference between the cases of day $i_{t}$ and $i_{{t - 1}}$, divided by the population of country *c* in 2019 (data from World Bank dataset^1^). We also computed *YCases pc*, the operationalization of $i_{{ct - 1}}$, the absolute value of cases at $t - 1$, divided once again by the population in order to obtain a per capita variable.

For lockdown measures, we relied on ACAPS data from the ‘COVID-19: Government Measures’ dataset. This allows us to distinguish between countries that applied lockdown measures, and countries that did not. We used the latest version available at the time of writing (the 24 March 2021 version), and built the dummy variable *Lockdown* (an operationalization of DLD), which assumes the value of 1 in the first date that a country implemented a partial or complete lockdown measure with respect to the entire population, and for all the subsequent days that the lockdown is in place. The choice to only include policies aimed at the entire population is justified in order to avoid having biased estimations due to policy interventions that were only referred to a small share of the population, which are likely to have a small impact on the total number of cases. This strategy results in a total of 264 measures captured by the DLD dummy, in 108 different countries, at many different times. DLD is also computed after a given number of days elapsed since the lockdown, namely on a daily basis, from 7 days after to 40 days after. 97.5% of those who develop symptoms do so within 11.5 days of infection, which means a confidence interval of between 8.2 and 15.6 days (Lauer et al., 2020): this suggests that the lockdown gives greater benefits in terms of a reduction in new cases only after the elapse of a certain time from the imposition of the policy.

Due to the listwise deletion of some minor Pacific islands and small European countries (such as Monaco, the Holy See and San Marino), and due to lack of data for the datasets of the other variables, 104 different countries are included in our final sample.

Finally, the last variable needed to perform our analysis is quality of government. This is proxied through the World Governance Indicator (Kaufmann et al., 2010) in its last available estimate (2018). More specifically, in order to test our research hypotheses we focused on three of the six dimensions^2^ proposed by Kaufmann that are more closely linked to the proposed mechanism: i) Government Effectiveness (GE), which captures perceptions of the quality of public services, the quality of the civil service and the degree of its independence from political pressures, the quality of policy formulation and implementation, and the credibility of the government's commitment to such policies; ii) Rule of Law (RL), which captures perceptions of the extent to which agents have confidence in and abide by the rules of society, and in particular the quality of contract enforcement, property rights, the police, and the courts, as well as the likelihood of crime and violence; and iii) Regulatory Quality (RQ), which captures perceptions of the ability of the government to formulate and implement sound policies and regulations that permit and promote private sector development (Kaufmann et al., 2010, p. 4). Following Andrés et al. (2015) these indexes can also be considered as variables for economic governance (with reference to regulatory quality and government effectiveness, which are proxies of the capacity of a government to formulate and implement policies, and to deliver services) and for institutional governance (with reference to rule of law, which is a proxy of respect for citizens and the state of institutions that govern the interactions among them).

Because the governance variables are computed annually (and thus are time invariant in our data, which cover the first wave of COVID-19, from 22 January to 31 May 2020), we cannot include them as covariates. For this reason, following the previously established approach in the literature (Alfano & Ercolano, 2020b), we take into account the differences between countries in terms of governance quality by splitting the sample into different subsamples on the basis of the world percentile rank of every country included in the analysis for each of these three dimensions.

Considering that data have several observations for each *c* and *t*, the best estimator to employ is a Feasible–Generalized Least Square (F-GLS) (Aigner & Balestra, 1988; Hsiao, 1986).

Moreover, given that the spread of the virus may be due to factors specific to each country, we consider it more appropriate to employ a Fixed Effects (FE) estimator, which captures heterogeneity between countries. In other words, we estimate the average effects with respect to the single countries, assuming that the heterogeneity among them does not change in the 130 days of our timespan. At the same time, this choice allows us to implicitly control for each variable that is time-invariant across the period examined (such as all those regarding demographics, the quality of the health systems, GDP per capita, cultural factors, and so on). Given the freshness of the topic, and the relatively scarce literature on the causes of COVID-19 spread, this seems to us to be the best approach to avoid having biased estimates due to omitted variables.

The final dataset, following the listwise deletion of some observations for which not all variables are available, as well as those countries that did not implement a lockdown, is composed of 130 daily observations (for the 130 days of the first wave, i.e. 22 January to 31 May) in 104 countries, giving a total of 13,520 observations. Other than from a theoretical perspective, in order to empirically test the appropriateness of a Fix Effects estimator, we performed an Hausman test on this sample, which confirmed that a Fix Effects estimate is to be preferred to a Random Effects one (the results are presented in Table 1).

**Table 1:** Hausman test

H0: difference in coefficients not systematic	
Chi2(2)	61.05
Prob > chi2	0.0000

It is worth bearing in mind that, given the impossibility in this empirical framework of including time-invariant variables as one of the covariates, our estimates will be split on the basis of the sample percentile rank (i.e., the rank considering all the country included in the analysis^3^) of every country included in the analysis for each of the three governance dimensions. For this reason, the resulting subsample of each quartile is composed of 26 countries, giving a total of 3380 observations per regression. It is also worth bearing in mind that different countries implemented lockdowns at different moment in time. Descriptive statistics of the variables are presented in Table 2.

**Table 2:** Summary statistics

Variable	Label	Obs	Mean	Std. Dev	Min	Max
New COVID-19 cases in day *t* over population	New cases pc	13,520	8.68e-06	.0000299	-.0002551	.0007566
COVID-19 cases in day *t* over population	Cases pc	13,520	.0003212	.0008812	0	.0065191
Seven day Moving Average of New COVID-19 cases in day *t* over population	MA New cases pc	13,520	8.19e-06	.0000244	-.0000195	.0002982
Dichotomous dummy variable (1 = lockdown in place)	dlkdwn	13,520	.4441568	.4968901	0	1
Dichotomous dummy variable (1 = lockdown in place for 10 days)	d10lkdwn	13,520	.3672337	.4820688	0	1
Dichotomous dummy variable (1 = lockdown in place for 20 days)	d20lkdwn	13,520	.2903107	.4539225	0	1
Dichotomous dummy variable (1 = lockdown in place for 25 days)	d25lkdwn	13,520	.2518491	.4340911	0	1
Dichotomous dummy variable (1 = lockdown in place for 30 days)	d30lkdwn	13,520	.2133876	.4097142	0	1
Dichotomous dummy variable (1 = lockdown in place for 40 days)	d40lkdwn	13,520	.1397929	.3467849	0	1

## Results

In order to estimate the varying impact of lockdowns in countries with different qualities of government, we run our model on subsamples. To test H1, we divide the sample for countries that are over the 75th percentile of Government Effectiveness (GE), and countries under the 25th (considering the whole WGI sample, not only the countries included in the analysis, in order to have an absolute quality measure and avoid sample selection biases). Similarly, H2 is tested by dividing the sample by Rule of Law (RL) percentiles, and H3 by those for Regulatory Quality (RQ). Before discussing the results, it is important to note that while the choice of the threshold of countries under the 25th percentile and over the 75th may seem arbitrary, a number of robustness checks (not included for brevity, but available from the authors upon request) suggest the solidity of this result using different thresholds, such as the median.

**Fig. 1:**
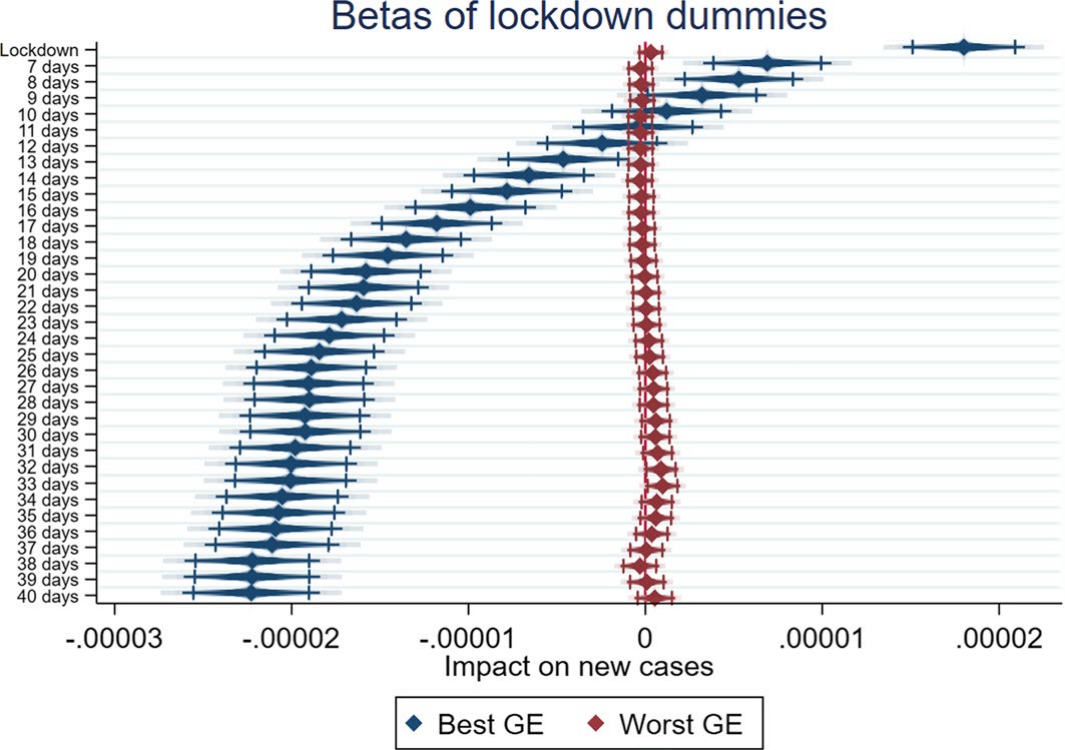
Government Effectiveness, Lockdown effects—best and worst quartiles compared. Diamonds represent beta estimates; dark lines 95% confidence interval; light lines 90% confidence interval

**Fig. 2:**
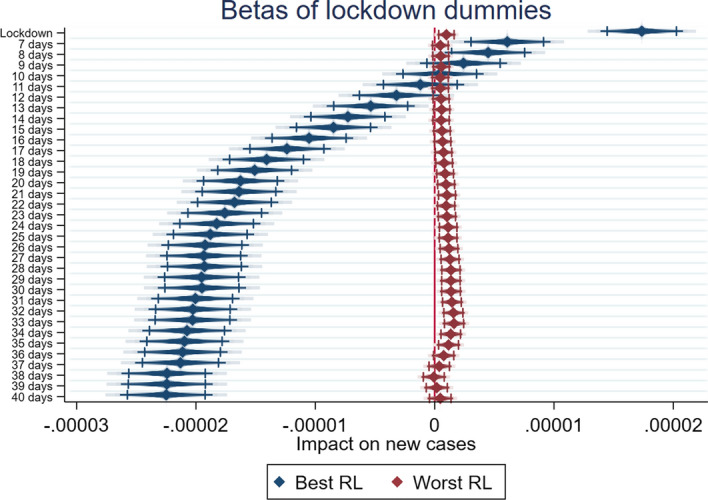
Rule of Law, Lockdown effects—best and worst quartiles compared. Diamonds represent beta estimates; dark lines 95% confidence interval; light lines 90% confidence interval

**Fig. 3:**
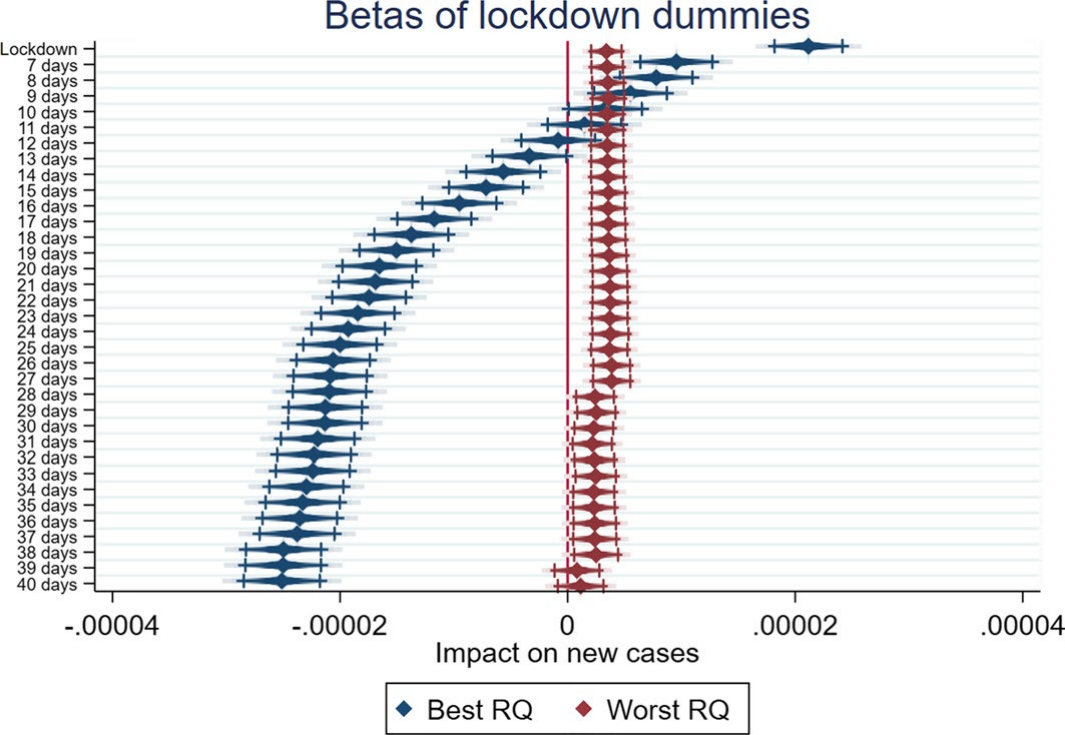
Regulatory quality, Lockdown effects—best and worst quartiles compared. Diamonds represent beta estimates; dark lines 95% confidence interval; light lines 90% confidence interval

Looking at the results, there seems to be confirmation for H1, H2 and H3, as summarized by Figs. 1, 2 and 3, which report the coefficients of the various lockdown dummies in the different specifications for the two subsamples, and show the dynamic over time: countries in the best quartile for WGI (in blue) have better performances in the reduction of per capita new COVID-19 cases than countries from the worst quartile (in red), which have little or no statistically significant benefit from the lockdowns.

More specifically, in Tables 3 and 4 we report the results related to the subsample divided on the basis of GE. Looking at the best quartile (Table 3), the results show that starting from 20 days after lockdown, the negative coefficients appear statistically significant, with an increase in magnitude up to 40 days. The coefficients related to the worst quartile of GE are not statistically significant for the threshold that is typically considered, with the exception of the dummy for lockdowns that have lasted over 30 days, which shows a positive sign. On the basis of the comparison between the best and worst quartiles of GE we can conclude that our results confirm H1. Countries characterized by greater confidence in the quality of policy formulation, along with the government’s commitment to such policies, implement more effective lockdowns.

**Table 3:** F-GLS estimation with fixed effects. Best quartile for Government Effectiveness

	(1)	(2)	(3)	(4)	(5)	(6)
	New Cases pc	New Cases pc	New Cases pc	New Cases pc	New Cases pc	New Cases pc
YCases pc	0.00406***	0.00806***	0.0130***	0.0136***	0.0140***	0.0131***
	(5.73)	(10.66)	(17.50)	(19.02)	(20.35)	(20.54)
Dummy Lockdown	0.0000206***					
	(11.94)					
Dummy Lockdown 10 days		0.00000528***				
		(2.83)				
Dummy Lockdown 20 days			− 0.0000131***			
			(− 6.92)			
Dummy Lockdown 25 days				− 0.0000168***		
				(− 8.88)		
Dummy Lockdown 30 days					− 0.0000202***	
					(− 10.65)	
Dummy Lockdown 40 days						− 0.0000212***
						(− 10.75)
Constant	0.00000517***	0.0000100***	0.0000128***	0.0000129***	0.0000128***	0.0000123***
	(5.54)	(11.28)	(15.23)	(15.63)	(15.81)	(15.29)
Observations	3380	3380	3380	3380	3380	3380
Rho	0.133	0.0902	0.0568	0.0535	0.0517	0.0553
R2	0.135	0.147	0.184	0.193	0.202	0.201

*t* statistics in parentheses

*p < 0.1; **p < 0.05; ***p < 0.01

**Table 4:** F-GLS estimation with fixed effects. Worst quartile for Government Effectiveness

	(1)	(2)	(3)	(4)	(5)	(6)
	New Cases pc	New Cases pc	New Cases pc	New Cases pc	New Cases pc	New Cases pc
YCases pc	0.0459***	0.0463***	0.0458***	0.0456***	0.0454***	0.0459***
	(42.29)	(42.50)	(42.01)	(41.82)	(41.76)	(42.98)
Dummy Lockdown	0.000000308					
	(0.78)					
Dummy Lockdown 10 days		− 0.000000292				
		(− 0.70)				
Dummy Lockdown 20 days			0.000000375			
			(0.83)			
Dummy Lockdown 25 days				0.000000749		
				(1.56)		
Dummy Lockdown 30 days					0.00000109**	
					(2.09)	
Dummy Lockdown 40 days						0.000000629
						(0.98)
Constant	0.000000164	0.000000364	0.000000196	0.000000142	0.000000119	0.000000224
	(0.70)	(1.64)	(0.93)	(0.69)	(0.59)	(1.16)
Observations	3380	3380	3380	3380	3380	3380
Rho	0.00443	0.00388	0.00448	0.00482	0.00511	0.00451
R2	0.457	0.457	0.457	0.457	0.457	0.457

*t* statistics in parentheses

*p < 0.1, ** *p* < 0.05, *** *p* < 0.01

A similar conclusion may be reached by looking at the results related to the subsample divided on the basis of RL. In this case too the best quartile (Table 5) starts to show the expected negative sign from 20 days after lockdown, with an increase of the magnitude up to 40 days after the implementation of lockdown. The results of the worst quartile in terms of the RL indicator (Table 6) show more statistically significant dummy variables, although, once again, with a positive and growing sign up to 30 days after lockdown. This finding confirms our H2. A country’s capacity to ensure the rules of society, which is an incentive for the population to respect the lockdown, fosters the effectiveness of lockdown.

**Table 5:** F-GLS estimation with fixed effects. Best quartile for Rule of Law

	(1)	(2)	(3)	(4)	(5)	(6)
	New Cases pc	New Cases pc	New Cases pc	New Cases pc	New Cases pc	New Cases pc
YCases pc	0.00422***	0.00826***	0.0131***	0.0137***	0.0141***	0.0131***
	(5.94)	(10.94)	(17.69)	(19.16)	(20.44)	(20.57)
Dummy Lockdown	0.0000200***					
	(11.56)					
Dummy Lockdown 10 days		0.00000450**				
		(2.42)				
Dummy Lockdown 20 days			− 0.0000136***			
			(− 7.21)			
Dummy Lockdown 25 days				− 0.0000172***		
				(− 9.11)		
Dummy Lockdown 30 days					− 0.0000204***	
					(− 10.82)	
Dummy Lockdown 40 days						− 0.0000214***
						(− 10.87)
Constant	0.00000525***	0.0000101***	0.0000128***	0.0000129***	0.0000128***	0.0000122***
	(5.62)	(11.33)	(15.21)	(15.58)	(15.75)	(15.22)
Observations	3380	3380	3380	3380	3380	3380
Rho	0.132	0.0894	0.0570	0.0539	0.0523	0.0560
R2	0.135	0.148	0.186	0.195	0.204	0.202

*t* statistics in parentheses

*p < 0.1; **p < 0.05; ***p < 0.01

**Table 6:** F-GLS estimation with fixed effects. Worst quartile for Rule of Law

	(1)	(2)	(3)	(4)	(5)	(6)
	New Cases pc	New Cases pc	New Cases pc	New Cases pc	New Cases pc	New Cases pc
YCases pc	0.0386***	0.0389***	0.0385***	0.0384***	0.0383***	0.0389***
	(46.16)	(46.09)	(45.43)	(45.18)	(44.94)	(46.07)
Dummy Lockdown	0.000000994**					
	(2.50)					
Dummy Lockdown 10 days		0.000000478				
		(1.15)				
Dummy Lockdown 20 days			0.00000103**			
			(2.32)			
Dummy Lockdown 25 days				0.00000125***		
				(2.67)		
Dummy Lockdown 30 days					0.00000155***	
					(3.10)	
Dummy Lockdown 40 days						0.000000535
						(0.91)
Constant	0.000000185	0.000000431*	0.000000330	0.000000323	0.000000316	0.000000527***
	(0.74)	(1.85)	(1.50)	(1.50)	(1.51)	(2.62)
Observations	3380	3380	3380	3380	3380	3380
Rho	0.0179	0.0168	0.0180	0.0184	0.0190	0.0167
R2	0.512	0.512	0.512	0.512	0.512	0.512

*t* statistics in parentheses

*p < 0.1; **p < 0.05; ***p < 0.01

Finally, in Tables 7 and 8 we report the results of the analysis performed on the best and worst quartiles based on the RQ indicator. In this case too, countries belonging to the best quartile start to show the expected negative sign from 20 days after the implementation of lockdown. Moreover, the magnitude of the coefficients grows and keeps its statistical significance up to 40 days after lockdown. On the other hand, countries belonging to the worst quartile for the RQ indicator show a positive coefficient for the lockdown dummies. These results confirm H3: countries where the government is perceived as having the capacity to implement sound policies can implement more effective lockdowns.

**Table 7:** F-GLS estimation with fixed effects. Best quartile for Regulatory Quality

	(1)	(2)	(3)	(4)	(5)	(6)
	New Cases pc	New Cases pc	New Cases pc	New Cases pc	New Cases pc	New Cases pc
YCases pc	0.00358***	0.00799***	0.0131***	0.0137***	0.0139***	0.0128***
	(5.00)	(10.48)	(17.67)	(19.15)	(20.40)	(20.34)
Dummy Lockdown	0.0000209***					
	(11.59)					
Dummy Lockdown 10 days		0.00000419**				
		(2.15)				
Dummy Lockdown 20 days			− 0.0000152***			
			(− 7.73)			
Dummy Lockdown 25 days				− 0.0000187***		
				(− 9.59)		
Dummy Lockdown 30 days					− 0.0000219***	
					(− 11.23)	
Dummy Lockdown 40 days						− 0.0000222***
						(− 10.98)
Constant	0.00000645***	0.0000112***	0.0000137***	0.0000137***	0.0000135***	0.0000130***
	(6.85)	(12.47)	(16.04)	(16.30)	(16.37)	(15.80)
Observations	3380	3380	3380	3380	3380	3380
Rho	0.130	0.0849	0.0532	0.0507	0.0497	0.0537
R2	0.124	0.140	0.179	0.188	0.196	0.193

*t* statistics in parentheses

*p < 0.1; **p < 0.05; ***p < 0.01

**Table 8:** F-GLS estimation with fixed effects. Worst quartile for Regulatory Quality

	(1)	(2)	(3)	(4)	(5)	(6)
	New Cases pc	New Cases pc	New Cases pc	New Cases pc	New Cases pc	New Cases pc
YCases pc	0.0197***	0.0190***	0.0179***	0.0175***	0.0183***	0.0198***
	(12.02)	(11.38)	(10.47)	(10.07)	(10.35)	(10.91)
Dummy Lockdown	0.00000372***					
	(4.56)					
Dummy Lockdown 10 days		0.00000426***				
		(4.94)				
Dummy Lockdown 20 days			0.00000540***			
			(5.68)			
Dummy Lockdown 25 days				0.00000578***		
				(5.67)		
Dummy Lockdown 30 days					0.00000462***	
					(4.14)	
Dummy Lockdown 40 days						0.00000299**
						(2.17)
Constant	7.37e−08	0.000000222	0.000000320	0.000000467	0.000000856**	0.00000128***
	(0.15)	(0.47)	(0.72)	(1.08)	(2.01)	(3.12)
Observations	3380	3380	3380	3380	3380	3380
Rho	0.0170	0.0177	0.0188	0.0192	0.0182	0.0169
R2	0.0950	0.0954	0.0964	0.0960	0.0930	0.0909

*t* statistics in parentheses

*p < 0.1; **p < 0.05; ***p < 0.01

By comparing the different dynamics for the different subsamples, it seems clear that RQ is the most important indicator in determining an efficient lockdown. Indeed, countries from the top quartile perform exceptionally well compared to countries from the bottom quartile. The second most important governance indicator for predicting the efficiency of a lockdown is RL. Finally, while countries from the top quartile for GE also show better effects from lockdown than countries from the bottom quartile, the difference is not as big as when the sample is divided by the other two dimensions. Nevertheless, it is worth noting that RQ is the measure that is most closely linked to the concept of policy effectiveness. GE directly measures the quality of public service supplied by a government, while RL captures the quality of the institutional setting. RQ and RL measure the perceptions regarding the ability of the government to formulate and implement sound policies. The underlined mechanism could suggest that more than the perception of good governments and public services (proxied by GE) able to promote trust, or a proper institutional setting (proxied by RL), the perception of a good policy formulation (proxied by RQ) positively impacts on the effectiveness of stringent measures like lockdowns. This finding contributes to the cited literature, adding some information about the relationship between governance quality and individual confidence in public services, and especially in health services (Chan et al., 2020). Indeed, countries characterized by confidence in the provision of public goods and services (such as healthcare), proxied by GE, show the expected negative sign, but its magnitude is relatively small compared to the sample split on the basis of the dimensions able to synthetize the quality of policy, proxied by RQ.

Moreover, if we look at the countries belonging to the worst quartile, the lack of a statistically significant effect, or in some cases the positive sign associated to the lockdown dummies, may suggest some interesting implications. This result enriches the findings of the literature on the effectiveness of lockdowns (Alfano & Ercolano, 2020a; Riccardo et al., 2020; Sardar et al., 2020; Shao, 2020), adding an important piece to a very complex puzzle that highlights how governance quality, and especially the perception of the quality of policies, matters in fostering effective lockdowns.

It may be tempting to justify these results by referring to other characteristics of the countries that are not included in the analysis and which are highly correlated with the WGI (such as the quality of the health system). As a matter of fact, when dividing the sample by WGI one is also dividing by the underlying variables that are highly correlated with them. Nevertheless, our analysis focuses on the spread of the infection (i.e., our dependent variables are the new cases of COVID-19), and thus it is difficult to imagine that these underlying variables, which may indeed play a role in determining death rates or other variables determined by policies directly aimed at strengthening the hospital system, also play a role in this context.

Moreover, despite the centrality of the healthcare system in handling the pharmaceutical response, the COVID-19 pandemic is substantially more complex than a merely pharmaceutical crisis, and it is obvious that in order to be fought effectively it requires a wider range of measures. This peculiarity of the COVID-19 pandemic can partially explain why governance quality factors may matter more in explaining the effectiveness of such policies.

## Robustness Check

To further test the importance of WGI in the effectiveness of lockdown, we designed and performed a number of robustness checks. First, we considered whether our dependent variable could be affected by some measurement error. COVID-19 cases are not always correctly reported and identified, and this issue is especially pronounced when one takes a number of countries into account over a substantial period of time, due to differences in the testing policy of different countries, as well as the improvement of this process over time (and, of course, of the varying opportunity for people to access tests and for them and the relative data to be processed without human errors). Unfortunately, there is not much that is possible to do to correct these biases; nonetheless, they should be normally distributed with the exception of the bias that is due to optimization of the procedure over time. For this reason, as a robustness test we chose to replicate our analysis using as dependent variable *MA New Cases pc*, i.e., the seven-day moving average of New Cases, our operationalization of new daily COVID-19 cases per capita. This strategy allows us to smooth the series over time, and to reduce the effect of errors in point estimates on our overall results. This robustness check presents equal results to our principal results: regressions are included in Tables SM1a to SM3b of the supplementary materials.

Second, as a further robustness check we replicated the analysis using a random effects estimator rather than a fix effects estimator. Despite what the Hausman test and our theoretical framework suggest, this relationship is worth exploring from a between-country (Random effects) as well as a within-country (Fix effects) point of view. The results, which are once again equivalent to those presented above, are available in Tables SM4a to SM6b of the supplementary materials.

The random effects estimation is especially interesting, since it allows us to overcome the main limitation of a fix effects estimation, which is the impossibility of including time-invariant variables. As a further robustness check, we exploit this possibility, modifying our model by including an interaction term between each of the WGI scores and the lockdown variables. This allows us to evaluate the impact on new COVID-19 cases of the WGI performances once a lockdown is in place, and after a given number of days a lockdown is in place.

To better exploit this possibility, we also run a model adding a new term DLD*WGI, which is equal to the result of each operationalization of *WGI* with the various lockdown variables. In more formal terms, in order to explore the impact of *WGI* on lockdowns, we modified Eq. (1) as:

2 $$ \Delta i_{{ct}}  = \alpha  + \beta _{1} i_{{ct - 1}}  + \beta _{2} DLD_{{ct}}  + \beta _{3} WGI_{{ct}}  + \beta _{4} DLD_{{ct}} *WGI_{{ct}}  + \varepsilon $$

where *WGI* is the value in the last available edition of one of the three World Governance Indicator variables included in the analysis, and WGI*DLD is the interaction term between the WGI studied and the DLD.

**Fig. 4:**
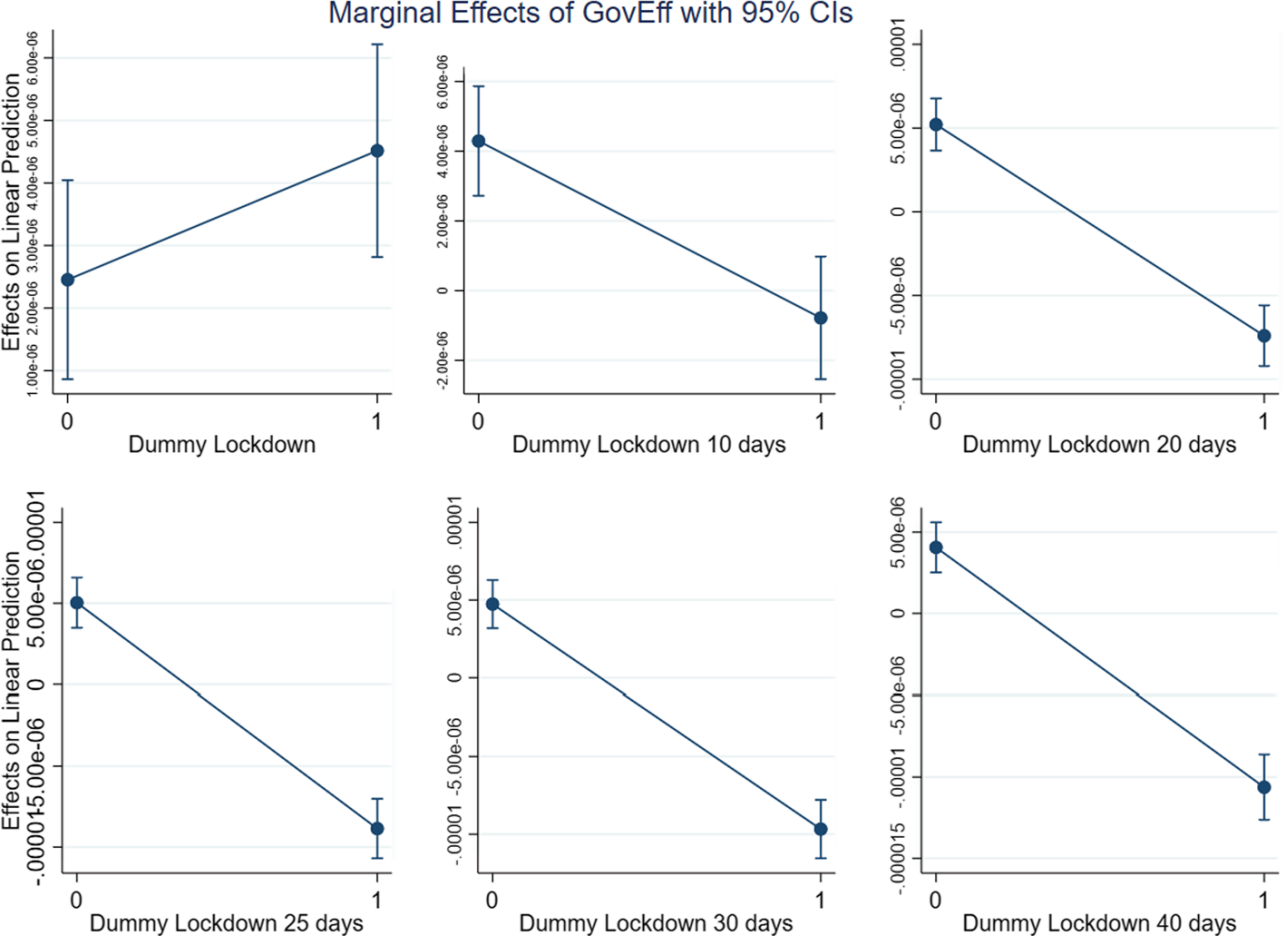
Marginal effects of Government Effectiveness on New Cases pc for the different lockdown dummies

**Fig. 5:**
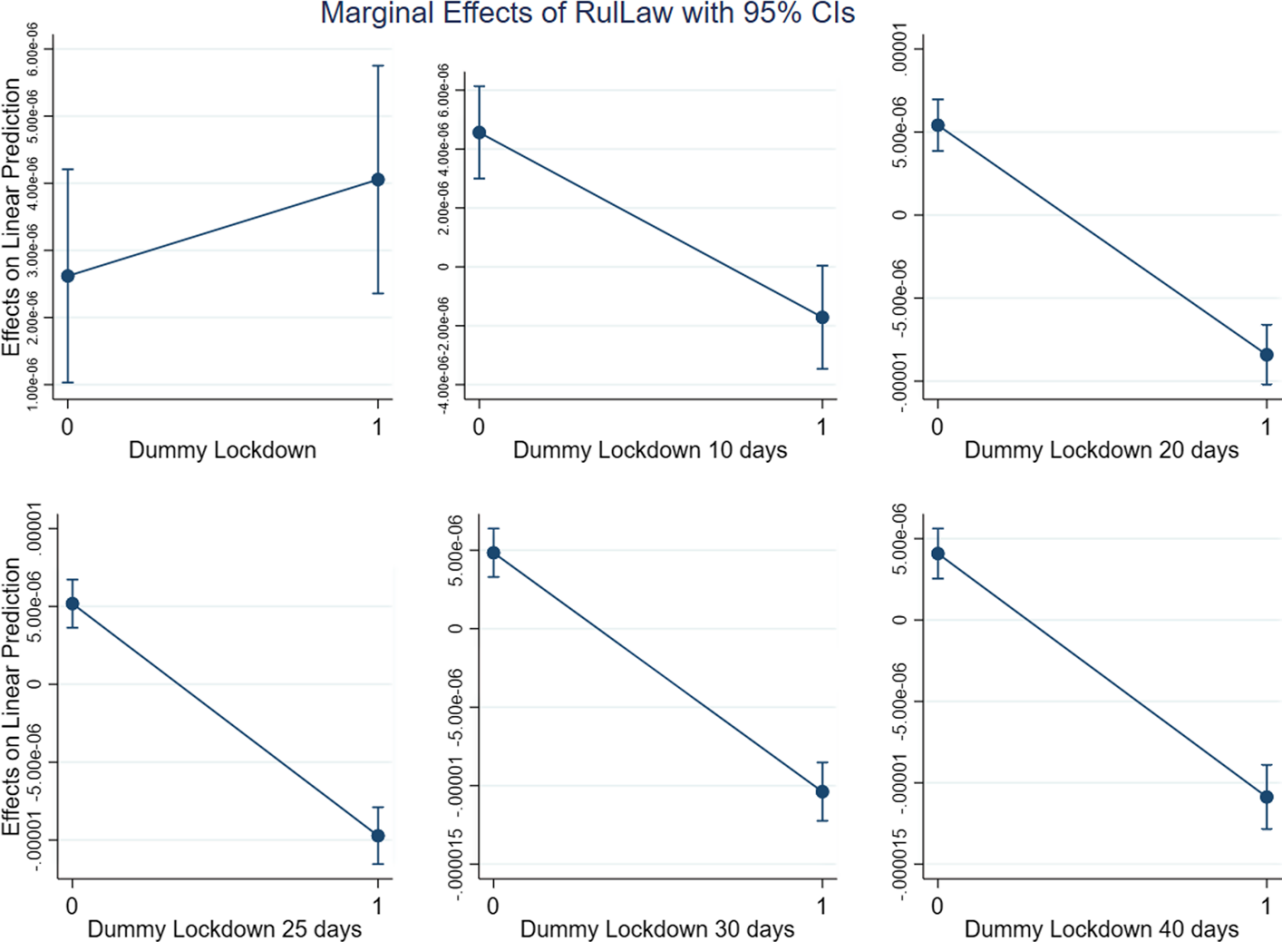
Marginal effects of Rule of Law on New Cases pc for the different lockdown dummies

**Fig. 6:**
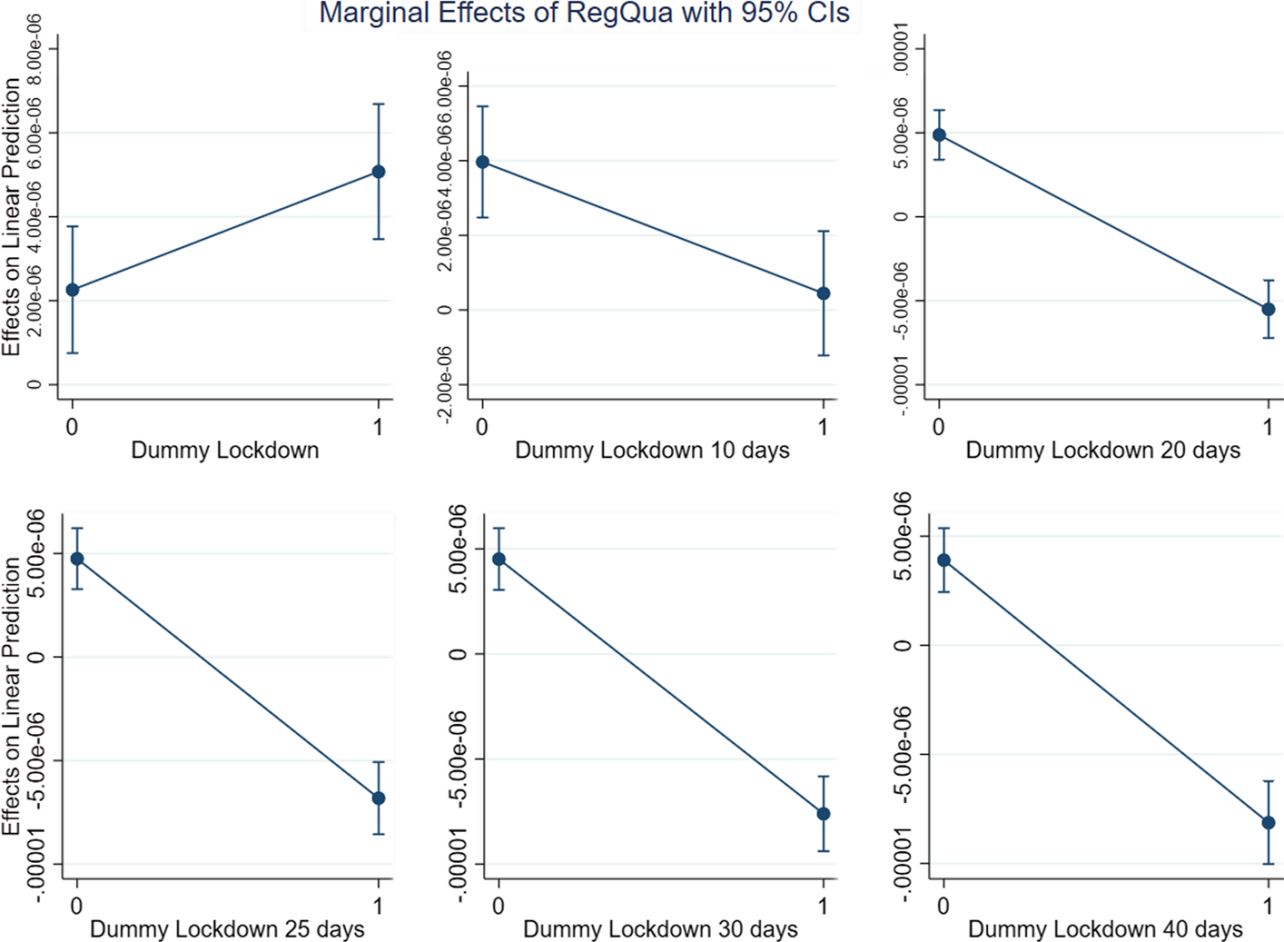
Marginal effects of Regulatory Quality on New Cases pc for the different lockdown dummies

**Fig. 7:**
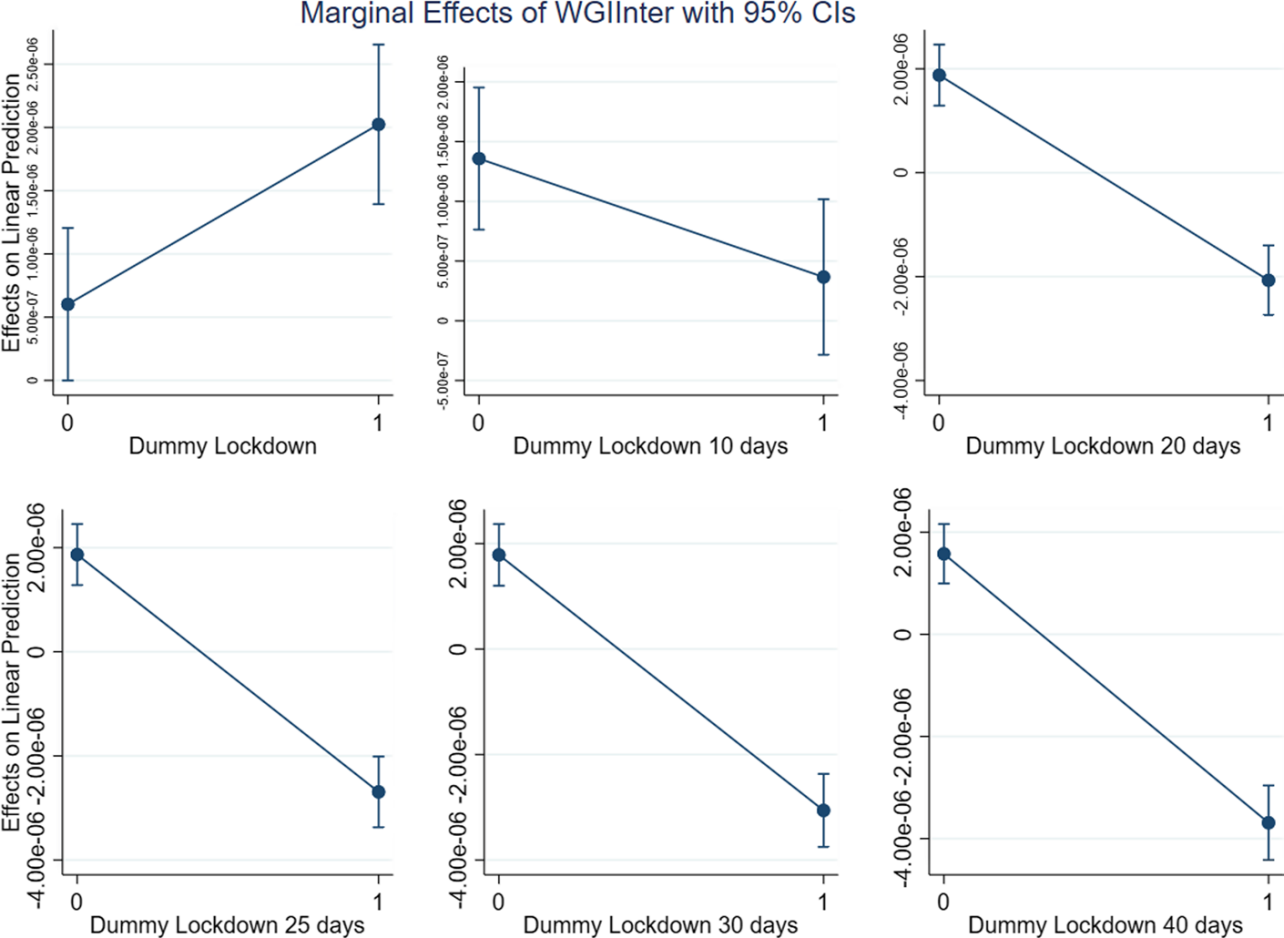
Marginal effects of WGIInter on New Cases pc for the different lockdown dummies

Given that (2) includes an interaction term, the impact of WGI on the different levels of DLD (which, as a dummy variable signalling the presence of a lockdown, may assume two values only) can be observed by computing the marginal effect. This is what we plot in Figs. 4, 5, 6 and 7, with one for each of the three dimensions of WGI studied in this paper (GE, RL and RQ). We also included a new variable, *WGIinter*, which is equal to the interaction of the three WGI indexes. In more formal terms:

$$ WGIInter = GE*RL*RQ $$

This allows us to compute the impact of all the WGI dimensions on lockdown efficacy.

As can be seen in Figs. 4, 5, 6 and 7, which show the marginal effects of WGI on the dependent variable, the dynamic is common to each of the four operationalizations of WGI: immediately after a lockdown the impact of *WGI* on the dependent variable *New Cases pc* is positive. This is probably due to the fact that the virus has a more severe impact in more developed countries, where people are more mobile and where the contagion accordingly spreads faster. Ten days after the lockdown, we observe that the effect of *WGI* on *New Cases pc* becomes negative, and its magnitude continues grow over time (i.e., the line steepens). This is exactly what we expect, since the effect of lockdowns on the number of new cases is not immediate, but on the contrary requires some time to be observed (Alfano & Ercolano, 2020a). If a lockdown is in place for at least 20 days, for each of the WGI variables (and for *WGIInter*, their interaction) the marginal effect becomes significantly negative, suggesting that the WGI variables do indeed play a role in reducing new COVID-19 cases once a lockdown is in place.

## Conclusion

For governments, the decision whether or not to implement lockdown measures is a very difficult choice given the associated economic costs, but also due to the individual costs related to a severe restriction of freedom that people are not accustomed to. Because of this, it is reasonable to think that citizens’ perception of their government’s capacity to plan and implement sound policies, measured according to the “governance” approach proposed by Kaufmann et al. (2010), could affect the effectiveness of lockdown.

This study provides the first empirical evidence from a cross-country perspective of the impact of governance dimension on the efficacy of lockdown measures. Our results confirm that Regulatory Quality, Rule of Law and Government Effectiveness have a positive impact on the efficiency of lockdowns. These findings shed some light on hidden benefits related to better institutional environments, which are able to affect citizens’ compliance positively even in the presence of very restrictive policies.

Moreover, these results further the debate on defining “optimal lockdown”, “mitigation measures” and “exit strategy”. Indeed, countries that perform better in these three governance dimensions also perform better in lockdown effectiveness, thus reducing infection levels more quickly. It is important to highlight that ours is a cross-country analysis, and thus has to be considered as measuring average effects for the world and the different countries in the subsamples, with all the associated benefits in terms of generalization, but at the same time all the associated limits in deriving precise estimates. Caution is suggested in reading these results, which may also be partially driven by the timing of the measures in Europe and the rest of the world, as well as the spread of the pandemic.

For this reason, we highlight the importance of and need for further investigation of this topic. First of all, in order to overcome some of the possible limitations of the present study, further validation could arise from subsequent analysis based on regional data or more restricted samples.

Moreover, it is worth noting that one may consider our results not to be fully generalizable as regards the issue of policy effectiveness, due to the specificity of the historical moment; however, as pointed out by some scholars (Chan et al., 2020), it is precisely the uniqueness of the COVID-19 pandemic, characterized by the random nature of the shock as well as its global dimension, that deepens our knowledge of the complex relations between compliance and policy effectiveness. On this specific point, an interesting further investigation should be devoted to better isolating how confidence and trust can be correlated with the effectiveness of lockdown measures.

## Supplementary Information

Below is the link to the electronic supplementary material.

Supplementary file1 (DOCX 58 kb)

## Footnotes

1 With the exception of Eritrea, which is not available in the World Bank dataset, and whose population is estimated at 3,580,551, according to the Worldometer elaboration.

2 This choice is justified due to greater relevance on a theoretical basis for the dimension chosen. Indeed, there is no specific reason why Control of Corruption, Voice and Accountability or Political Stability and Absence of Violence should have an impact on the effectiveness of implementing a lockdown policy aimed at the whole population.

3 Please note that as a robustness check we also replicate the analysis splitting the sample by percentiles, and considering the whole WGI dataset, i.e., not limiting it to the sample. The regression, not included for reasons of space but available from the authors upon request, shows equal results.

## References

[CR1] Alabede JO, Zainal Affrin Z (2011). Individual taxpayers’ attitude and compliance behaviour in Nigeria: The moderating role of financial condition and risk preference. Journal of Accounting and Taxation.

[CR2] Alfano V, Ercolano S (2020). The Efficacy of Lockdown Against COVID-19: A Cross-Country Panel Analysis. Applied Health Economics and Health Policy.

[CR3] Alfano V, Ercolano S (2020). Bonding and Bridging Social Capital and Lockdown. An Analysis of the Italian Regions. Rivista Economica Del Mezzogiorno.

[CR4] Alfano V, Sgobbi M (2021). A peste, fame et bello libera nos, Domine. Journal of Family History.

[CR501] Aigner, D. J., & Balestra, M. (1988). Optimal experimental design for error components models. *Econometrica, 56*(4), 955–971.

[CR5] Andrés RA, Asongu SA, Amavilah VH (2015). The Impact of Formal Institutions on Knowledge Economy. Journal of the Knowledge Economy.

[CR6] Bargain O, Aminjonov U (2020). Trust and compliance to public health policies in times of COVID-19. Journal of Public Economics.

[CR7] Bickley, S. J., Chan, H. F., Skali, A., Stadelmann, D., & Torgler, B. (2020). How does globalisation affect COVID-19 responses?. 10.21203/rs.3.rs-39311/v2 10.1186/s12992-021-00677-5 PMC8134968 34016146

[CR8] Chan HF, Brumpton M, Macintyre A, Arapoc J, Savage DA, Skali A, Stadelmann D, Torgler B (2020). How confidence in health care systems affects mobility and compliance during the COVID-19 pandemic. PLoS ONE.

[CR9] Dong E, Du H, Gardner L (2020). An interactive web-based dashboard to track COVID-19 in real time. The Lancet Infectious Diseases.

[CR10] Hamzelou J (2020). World in lockdown. New Scientist.

[CR503] Hsiao, C. (1986). *Analysis of panel data*. Cambridge: Cambridge University Press.

[CR11] Kaufmann, D., Kraay, A. & Mastruzzi, M. (2010). The Worldwide Governance Indicators: Methodology and Analytical Issues (September 2010). World Bank Policy Research Working Paper No. 5430.

[CR12] Knack S, Zak PJ (2003). Building trust: Public policy, interpersonal trust, and economic development. Supreme Court Economic Review.

[CR13] Koppell JG, Auer JC (2012). Is There a Spirit of Governance?. Public Administration Review.

[CR14] Lau H, Khosrawipour V, Kocbach P, Mikolajczyk A, Schubert J, Bania J (2020). The positive impact of lockdown in Wuhan on containing the COVID-19 outbreak in China. Journal of Travel Medicine.

[CR505] Lauer, S. A., Grantz, K. H., Bi, Q., Jones, F. K., Zheng, Q., Meredith, H., et al. (2020). The incubation period of coronavirus disease 2019 (COVID-19) from publicly reported confirmed cases: Estimation and application. *Annals of Internal Medicine, 172*(9), 577–582. 10.7326/M20-0504 PMC7081172 32150748

[CR15] Letki N (2006). Investigating the roots of civic morality: Trust, social capital, and institutional performance. Political Behavior.

[CR16] Ljunge M (2014). Social capital and political institutions: Evidence that democracy fosters trust. Economics Letters.

[CR17] Marien S, Hooghe M (2011). Does political trust matter? An empirical investigation into the relation between political trust and support for law compliance. European Journal of Political Research.

[CR18] Munster VJ, Koopmans M, van Doremalen N, van Riel D, de Wit E (2020). A Novel Coronavirus Emerging in China—Key Questions for Impact Assessment. The New England Journal of Medicine.

[CR19] Perry JL, de Graaf G, van der Wal Z, van Montfort C (2014). Returning to our roots: “Good government” evolves to “good governance”. Public Administration Review.

[CR20] Piguillem, F., & Shi L. (2020) The optimal covid-19 quarantine and testing policies (No. 2004). Einaudi Institute for Economics and Finance (EIEF).

[CR500] Ramírez de la Cruz, E. E., Grin, E. J., Sanabria-Pulido, P., Cravacuore, D., & Orellana, A. (2020) The transaction costs of government responses to the COVID-19 emergency in Latin America. *Public Administration Review, 80*(4), 683–695. 10.1111/puar.13259 PMC7300791 32836458

[CR21] Riccardo F., Ajelli M., Andrianou X., Bella A., Del Manso M., Fabiani M., et al. (2020) Epidemiological characteristics of COVID-19 cases in Italy and estimates of the reproductive numbers one month into the epidemic. 2020. https://www.medrxiv.org/content/10.1101/2020.04.08.20056861v1. 10.2807/1560-7917.ES.2020.25.49.2000790 PMC7730489 33303064

[CR22] Rothe C, Schunk M, Sothmann P, Bretzel G, Froeschl G, Wallrauch C, Zimmer T, Thiel V, Janke C, Guggemos W, Seilmaier M, Drosten C, Vollmar P, Zwirglmaier K, Zange S, Wölfel R, Hoelscher M (2020). Transmission of 2019-nCoV Infection from an Asymptomatic Contact in Germany. The New England Journal of Medicine.

[CR23] Sardar, T., Nadim, S. S. & Chattopadhyay, J. (2020) Assessment of 21 days lockdown effect in some states and overall India: a predictive mathematical study on COVID-19 outbreak. Available at: https://arxiv.org/abs/2004.03487 10.1016/j.chaos.2020.110078 PMC7345298 32834620

[CR24] Shao, P. (2020). Impact of city and residential unit lockdowns on prevention and control of COVID-19. Available at: https://www.medrxiv.org/content/10.1101/2020.03.13.20035253v1.

[CR25] Torgler, B., Schaffner, M., & Macintyre, A. (2007). Tax compliance, tax morale and governance quality (No. 2007–17). CREMA Working Paper.

[CR26] Torgler B, Schneider F (2009). The impact of tax morale and institutional quality on the shadow economy. Journal of Economic Psychology.

[CR27] Toshkov, D., Yesilkagit, K., & Carroll, B. (2020). Government capacity, societal trust or party preferences? What accounts for the variety of national policy responses to the COVID-19 pandemic in Europe?

[CR28] Umar MA, Derashid C, Ibrahim I, Bidin Z (2019). Public governance quality and tax compliance behavior in developing countries: the mediating role of socioeconomic conditions. International Journal of Social Economics.

[CR29] Zak PJ, Knack S (2001). Trust and growth. The Economic Journal.

