# Supplementary material for: Stay at Home! Governance Quality and Effectiveness of Lockdown
Source: Soc Indic Res. 2021 Jun 28;159(1):101–23. doi: 10.1007/s11205-021-02742-3 (PMC8238379; doi:10.1007/s11205-021-02742-3)
Supplement: Supplementary file 1 — Supplementary file1 (DOCX 58 kb) [file 11205_2021_2742_MOESM1_ESM.docx]

**Supplementary Material**

Table SM 1a - Best quartile per Government Effectiveness

|  | (1) | (2) | (3) | (4) | (5) | (6) |
| --- | --- | --- | --- | --- | --- | --- |
|  | M.A.New Cases pc | M.A.New Cases pc | M.A.New Cases pc | M.A.New Cases pc | M.A.New Cases pc | M.A.New Cases pc |
| YCases pc | 0.00689^***^ | 0.0101^***^ | 0.0153^***^ | 0.0164^***^ | 0.0167^***^ | 0.0158^***^ |
|  | (12.37) | (16.98) | (26.30) | (29.38) | (31.37) | (32.14) |
|  |  |  |  |  |  |  |
| Dummy Lockdown | 0.0000178^***^ |  |  |  |  |  |
|  | (13.08) |  |  |  |  |  |
|  |  |  |  |  |  |  |
| Dummy Lockdown 10 days |  | 0.00000532^***^ |  |  |  |  |
|  |  | (3.62) |  |  |  |  |
|  |  |  |  |  |  |  |
| Dummy Lockdown 20 days |  |  | -0.0000140^***^ |  |  |  |
|  |  |  | (-9.45) |  |  |  |
|  |  |  |  |  |  |  |
| Dummy Lockdown 25 days |  |  |  | -0.0000196^***^ |  |  |
|  |  |  |  | (-13.31) |  |  |
|  |  |  |  |  |  |  |
| Dummy Lockdown 30 days |  |  |  |  | -0.0000230^***^ |  |
|  |  |  |  |  | (-15.72) |  |
|  |  |  |  |  |  |  |
| Dummy Lockdown 40 days |  |  |  |  |  | -0.0000250^***^ |
|  |  |  |  |  |  | (-16.45) |
|  |  |  |  |  |  |  |
| Constant | 0.00000370^***^ | 0.00000771^***^ | 0.0000106^***^ | 0.0000109^***^ | 0.0000108^***^ | 0.0000102^***^ |
|  | (5.04) | (11.02) | (16.13) | (16.98) | (17.17) | (16.47) |
| Observations | 3380 | 3380 | 3380 | 3380 | 3380 | 3380 |
| Rho | 0.143 | 0.0979 | 0.0557 | 0.0509 | 0.0498 | 0.0528 |
| R2 | 0.256 | 0.262 | 0.306 | 0.325 | 0.339 | 0.342 |

*t* statistics in parentheses ^*^ *p* < 0.1, ^**^ *p* < 0.05, ^***^ *p* < 0.01

Table SM 1b - Worst quartile per Government Effectiveness

|  | (1) | (2) | (3) | (4) | (5) | (6) |
| --- | --- | --- | --- | --- | --- | --- |
|  | M.A.New Cases pc | M.A.New Cases pc | M.A.New Cases pc | M.A.New Cases pc | M.A.New Cases pc | M.A.New Cases pc |
| YCases pc | 0.0424^***^ | 0.0430^***^ | 0.0428^***^ | 0.0425^***^ | 0.0423^***^ | 0.0426^***^ |
|  | (94.18) | (94.91) | (94.37) | (93.77) | (93.53) | (95.97) |
|  |  |  |  |  |  |  |
| Dummy Lockdown | 0.000000178 |  |  |  |  |  |
|  | (1.09) |  |  |  |  |  |
|  |  |  |  |  |  |  |
| Dummy Lockdown 10 days |  | -0.000000470^***^ |  |  |  |  |
|  |  | (-2.73) |  |  |  |  |
|  |  |  |  |  |  |  |
| Dummy Lockdown 20 days |  |  | -0.000000261 |  |  |  |
|  |  |  | (-1.39) |  |  |  |
|  |  |  |  |  |  |  |
| Dummy Lockdown 25 days |  |  |  | 0.000000130 |  |  |
|  |  |  |  | (0.65) |  |  |
|  |  |  |  |  |  |  |
| Dummy Lockdown 30 days |  |  |  |  | 0.000000505^**^ |  |
|  |  |  |  |  | (2.34) |  |
|  |  |  |  |  |  |  |
| Dummy Lockdown 40 days |  |  |  |  |  | 4.89e-08 |
|  |  |  |  |  |  | (0.18) |
|  |  |  |  |  |  |  |
| Constant | 8.21e-08 | 0.000000286^***^ | 0.000000205^**^ | 0.000000124 | 7.41e-08 | 0.000000144^*^ |
|  | (0.84) | (3.10) | (2.35) | (1.46) | (0.89) | (1.78) |
| Observations | 3380 | 3380 | 3380 | 3380 | 3380 | 3380 |
| Rho | 0.0134 | 0.0127 | 0.0128 | 0.0133 | 0.0140 | 0.0132 |
| R2 | 0.798 | 0.799 | 0.798 | 0.798 | 0.798 | 0.798 |

*t* statistics in parentheses ^*^ *p* < 0.1, ^**^ *p* < 0.05, ^***^ *p* < 0.01

Table SM 2a - Best quartile per Rule of Law

|  | (1) | (2) | (3) | (4) | (5) | (6) |
| --- | --- | --- | --- | --- | --- | --- |
|  | M.A.New Cases pc | M.A.New Cases pc | M.A.New Cases pc | M.A.New Cases pc | M.A.New Cases pc | M.A.New Cases pc |
| YCases pc | 0.00703^***^ | 0.0103^***^ | 0.0154^***^ | 0.0165^***^ | 0.0168^***^ | 0.0158^***^ |
|  | (12.60) | (17.33) | (26.59) | (29.60) | (31.52) | (32.19) |
|  |  |  |  |  |  |  |
| Dummy Lockdown | 0.0000172^***^ |  |  |  |  |  |
|  | (12.67) |  |  |  |  |  |
|  |  |  |  |  |  |  |
| Dummy Lockdown 10 days |  | 0.00000457^***^ |  |  |  |  |
|  |  | (3.12) |  |  |  |  |
|  |  |  |  |  |  |  |
| Dummy Lockdown 20 days |  |  | -0.0000146^***^ |  |  |  |
|  |  |  | (-9.84) |  |  |  |
|  |  |  |  |  |  |  |
| Dummy Lockdown 25 days |  |  |  | -0.0000200^***^ |  |  |
|  |  |  |  | (-13.61) |  |  |
|  |  |  |  |  |  |  |
| Dummy Lockdown 30 days |  |  |  |  | -0.0000233^***^ |  |
|  |  |  |  |  | (-15.95) |  |
|  |  |  |  |  |  |  |
| Dummy Lockdown 40 days |  |  |  |  |  | -0.0000251^***^ |
|  |  |  |  |  |  | (-16.59) |
|  |  |  |  |  |  |  |
| Constant | 0.00000376^***^ | 0.00000777^***^ | 0.0000106^***^ | 0.0000109^***^ | 0.0000108^***^ | 0.0000102^***^ |
|  | (5.13) | (11.10) | (16.14) | (16.96) | (17.13) | (16.40) |
| Observations | 3380 | 3380 | 3380 | 3380 | 3380 | 3380 |
| Rho | 0.142 | 0.0969 | 0.0559 | 0.0513 | 0.0504 | 0.0536 |
| R2 | 0.256 | 0.263 | 0.308 | 0.328 | 0.341 | 0.344 |

*t* statistics in parentheses ^*^ *p* < 0.1, ^**^ *p* < 0.05, ^***^ *p* < 0.01

Table SM 2b - Worst quartile per Rule of Law

|  | (1) | (2) | (3) | (4) | (5) | (6) |
| --- | --- | --- | --- | --- | --- | --- |
|  | M.A.New Cases pc | M.A.New Cases pc | M.A.New Cases pc | M.A.New Cases pc | M.A.New Cases pc | M.A.New Cases pc |
| YCases pc | 0.0366^***^ | 0.0370^***^ | 0.0368^***^ | 0.0367^***^ | 0.0365^***^ | 0.0370^***^ |
|  | (92.79) | (92.74) | (91.92) | (91.31) | (90.88) | (92.62) |
|  |  |  |  |  |  |  |
| Dummy Lockdown | 0.000000693^***^ |  |  |  |  |  |
|  | (3.70) |  |  |  |  |  |
|  |  |  |  |  |  |  |
| Dummy Lockdown 10 days |  | 9.35e-08 |  |  |  |  |
|  |  | (0.48) |  |  |  |  |
|  |  |  |  |  |  |  |
| Dummy Lockdown 20 days |  |  | 0.000000269 |  |  |  |
|  |  |  | (1.28) |  |  |  |
|  |  |  |  |  |  |  |
| Dummy Lockdown 25 days |  |  |  | 0.000000544^**^ |  |  |
|  |  |  |  | (2.46) |  |  |
|  |  |  |  |  |  |  |
| Dummy Lockdown 30 days |  |  |  |  | 0.000000800^***^ |  |
|  |  |  |  |  | (3.40) |  |
|  |  |  |  |  |  |  |
| Dummy Lockdown 40 days |  |  |  |  |  | 8.45e-08 |
|  |  |  |  |  |  | (0.30) |
|  |  |  |  |  |  |  |
| Constant | 9.64e-08 | 0.000000346^***^ | 0.000000310^***^ | 0.000000261^**^ | 0.000000236^**^ | 0.000000367^***^ |
|  | (0.82) | (3.14) | (2.97) | (2.58) | (2.39) | (3.86) |
| Observation | 3380 | 3380 | 3380 | 3380 | 3380 | 3380 |
| Rho | 0.0320 | 0.0287 | 0.0296 | 0.0311 | 0.0324 | 0.0286 |
| R2 | 0.801 | 0.800 | 0.800 | 0.800 | 0.800 | 0.800 |

*t* statistics in parentheses ^*^ *p* < 0.1, ^**^ *p* < 0.05, ^***^ *p* < 0.01

Table SM 3a - Best quartile per Regulatory Quality

|  | (1) | (2) | (3) | (4) | (5) | (6) |
| --- | --- | --- | --- | --- | --- | --- |
|  | M.A.New Cases pc | M.A.New Cases pc | M.A.New Cases pc | M.A.New Cases pc | M.A.New Cases pc | M.A.New Cases pc |
| YCases pc | 0.00647^***^ | 0.0101^***^ | 0.0154^***^ | 0.0165^***^ | 0.0167^***^ | 0.0155^***^ |
|  | (11.40) | (16.65) | (26.46) | (29.55) | (31.42) | (31.82) |
|  |  |  |  |  |  |  |
| Dummy Lockdown | 0.0000179^***^ |  |  |  |  |  |
|  | (12.51) |  |  |  |  |  |
|  |  |  |  |  |  |  |
| Dummy Lockdown 10 days |  | 0.00000415^***^ |  |  |  |  |
|  |  | (2.69) |  |  |  |  |
|  |  |  |  |  |  |  |
| Dummy Lockdown 20 days |  |  | -0.0000163^***^ |  |  |  |
|  |  |  | (-10.52) |  |  |  |
|  |  |  |  |  |  |  |
| Dummy Lockdown 25 days |  |  |  | -0.0000218^***^ |  |  |
|  |  |  |  | (-14.28) |  |  |
|  |  |  |  |  |  |  |
| Dummy Lockdown 30 days |  |  |  |  | -0.0000250^***^ |  |
|  |  |  |  |  | (-16.49) |  |
|  |  |  |  |  |  |  |
| Dummy Lockdown 40 days |  |  |  |  |  | -0.0000263^***^ |
|  |  |  |  |  |  | (-16.73) |
|  |  |  |  |  |  |  |
| Constant | 0.00000482^***^ | 0.00000877^***^ | 0.0000114^***^ | 0.0000115^***^ | 0.0000113^***^ | 0.0000107^***^ |
|  | (6.47) | (12.31) | (16.93) | (17.58) | (17.63) | (16.84) |
| Observations | 3380 | 3380 | 3380 | 3380 | 3380 | 3380 |
| Rho | 0.139 | 0.0909 | 0.0520 | 0.0488 | 0.0485 | 0.0518 |
| R2 | 0.242 | 0.251 | 0.299 | 0.318 | 0.331 | 0.331 |

*t* statistics in parentheses ^*^ *p* < 0.1, ^**^ *p* < 0.05, ^***^ *p* < 0.01

Table SM 3b - Worst quartile per Regulatory Quality

|  | (1) | (2) | (3) | (4) | (5) | (6) |
| --- | --- | --- | --- | --- | --- | --- |
|  | M.A.New Cases pc | M.A.New Cases pc | M.A.New Cases pc | M.A.New Cases pc | M.A.New Cases pc | M.A.New Cases pc |
| YCases pc | 0.0272^***^ | 0.0269^***^ | 0.0265^***^ | 0.0262^***^ | 0.0266^***^ | 0.0283^***^ |
|  | (44.85) | (43.45) | (41.75) | (40.65) | (40.39) | (41.92) |
|  |  |  |  |  |  |  |
| Dummy Lockdown | 0.00000217^***^ |  |  |  |  |  |
|  | (7.16) |  |  |  |  |  |
|  |  |  |  |  |  |  |
| Dummy Lockdown 10 days |  | 0.00000241^***^ |  |  |  |  |
|  |  | (7.52) |  |  |  |  |
|  |  |  |  |  |  |  |
| Dummy Lockdown 20 days |  |  | 0.00000274^***^ |  |  |  |
|  |  |  | (7.78) |  |  |  |
|  |  |  |  |  |  |  |
| Dummy Lockdown 25 days |  |  |  | 0.00000303^***^ |  |  |
|  |  |  |  | (8.00) |  |  |
|  |  |  |  |  |  |  |
| Dummy Lockdown 30 days |  |  |  |  | 0.00000248^***^ |  |
|  |  |  |  |  | (5.98) |  |
|  |  |  |  |  |  |  |
| Dummy Lockdown 40 days |  |  |  |  |  | 0.000000116 |
|  |  |  |  |  |  | (0.22) |
|  |  |  |  |  |  |  |
| Constant | -0.000000131 | -2.13e-08 | 0.000000103 | 0.000000161 | 0.000000356^**^ | 0.000000702^***^ |
|  | (-0.70) | (-0.12) | (0.62) | (1.00) | (2.25) | (4.60) |
| Observations | 3380 | 3380 | 3380 | 3380 | 3380 | 3380 |
| Rho | 0.0664 | 0.0673 | 0.0685 | 0.0696 | 0.0673 | 0.0601 |
| R2 | 0.492 | 0.492 | 0.492 | 0.492 | 0.489 | 0.488 |

*t* statistics in parentheses ^*^ *p* < 0.1, ^**^ *p* < 0.05, ^***^ *p* < 0.01

Table SM 4a - Random Effects - Best quartile per Government Effectiveness

|  | (1) | (2) | (3) | (4) | (5) | (6) |
| --- | --- | --- | --- | --- | --- | --- |
|  | New Cases pc | New Cases pc | New Cases pc | New Cases pc | New Cases pc | New Cases pc |
| YCases pc | 0.00571^***^ | 0.00944^***^ | 0.0137^***^ | 0.0143^***^ | 0.0146^***^ | 0.0136^***^ |
|  | (8.43) | (13.22) | (19.63) | (21.04) | (22.26) | (22.37) |
|  |  |  |  |  |  |  |
| Dummy Lockdown | 0.0000178^***^ |  |  |  |  |  |
|  | (10.44) |  |  |  |  |  |
|  |  |  |  |  |  |  |
| Dummy Lockdown 10 days |  | 0.00000272 |  |  |  |  |
|  |  | (1.50) |  |  |  |  |
|  |  |  |  |  |  |  |
| Dummy Lockdown 20 days |  |  | -0.0000146^***^ |  |  |  |
|  |  |  | (-7.93) |  |  |  |
|  |  |  |  |  |  |  |
| Dummy Lockdown 25 days |  |  |  | -0.0000181^***^ |  |  |
|  |  |  |  | (-9.79) |  |  |
|  |  |  |  |  |  |  |
| Dummy Lockdown 30 days |  |  |  |  | -0.0000213^***^ |  |
|  |  |  |  |  | (-11.48) |  |
|  |  |  |  |  |  |  |
| Dummy Lockdown 40 days |  |  |  |  |  | -0.0000223^***^ |
|  |  |  |  |  |  | (-11.48) |
|  |  |  |  |  |  |  |
| Constant | 0.00000526^***^ | 0.00000996^***^ | 0.0000127^***^ | 0.0000127^***^ | 0.0000127^***^ | 0.0000120^***^ |
|  | (3.08) | (5.95) | (7.72) | (7.78) | (7.77) | (7.41) |
| Observations | 3380 | 3380 | 3380 | 3380 | 3380 | 3380 |
| Chi 2 | 474.5 | 358.7 | 425.0 | 461.1 | 500.1 | 500.4 |
| Rho | 0.0350 | 0.0334 | 0.0339 | 0.0342 | 0.0346 | 0.0344 |
| R2 | 0.149 | 0.153 | 0.184 | 0.193 | 0.202 | 0.201 |

*t* statistics in parentheses ^*^ *p* < 0.1, ^**^ *p* < 0.05, ^***^ *p* < 0.01

Table SM 4b - Random Effects - Worst quartile per Government Effectiveness

|  | (1) | (2) | (3) | (4) | (5) | (6) |
| --- | --- | --- | --- | --- | --- | --- |
|  | New Cases pc | New Cases pc | New Cases pc | New Cases pc | New Cases pc | New Cases pc |
| YCases pc | 0.0473^***^ | 0.0476^***^ | 0.0473^***^ | 0.0471^***^ | 0.0470^***^ | 0.0473^***^ |
|  | (51.75) | (51.95) | (51.58) | (51.46) | (51.45) | (52.44) |
|  |  |  |  |  |  |  |
| Dummy Lockdown | 0.000000126 |  |  |  |  |  |
|  | (0.33) |  |  |  |  |  |
|  |  |  |  |  |  |  |
| Dummy Lockdown 10 days |  | -0.000000449 |  |  |  |  |
|  |  | (-1.13) |  |  |  |  |
|  |  |  |  |  |  |  |
| Dummy Lockdown 20 days |  |  | 0.000000146 |  |  |  |
|  |  |  | (0.34) |  |  |  |
|  |  |  |  |  |  |  |
| Dummy Lockdown 25 days |  |  |  | 0.000000472 |  |  |
|  |  |  |  | (1.03) |  |  |
|  |  |  |  |  |  |  |
| Dummy Lockdown 30 days |  |  |  |  | 0.000000756 |  |
|  |  |  |  |  | (1.53) |  |
|  |  |  |  |  |  |  |
| Dummy Lockdown 40 days |  |  |  |  |  | 0.000000310 |
|  |  |  |  |  |  | (0.51) |
|  |  |  |  |  |  |  |
| Constant | 0.000000180 | 0.000000364^*^ | 0.000000195 | 0.000000138 | 0.000000112 | 0.000000200 |
|  | (0.77) | (1.65) | (0.93) | (0.68) | (0.56) | (1.04) |
| Observations | 3380 | 3380 | 3380 | 3380 | 3380 | 3380 |
| Chi 2 | 2839.9 | 2842.0 | 2839.9 | 2841.6 | 2844.0 | 2840.2 |
| Rho | 0 | 0 | 0 | 0 | 0 | 0 |
| R2 | 0.457 | 0.457 | 0.457 | 0.457 | 0.457 | 0.457 |

*t* statistics in parentheses ^*^ *p* < 0.1, ^**^ *p* < 0.05, ^***^ *p* < 0.01

Table SM 5a - Random Effects - Best quartile per Rule of Law

|  | (1) | (2) | (3) | (4) | (5) | (6) |
| --- | --- | --- | --- | --- | --- | --- |
|  | New Cases pc | New Cases pc | New Cases pc | New Cases pc | New Cases pc | New Cases pc |
| YCases pc | 0.00587^***^ | 0.00963^***^ | 0.0138^***^ | 0.0144^***^ | 0.0146^***^ | 0.0136^***^ |
|  | (8.65) | (13.49) | (19.82) | (21.17) | (22.35) | (22.40) |
|  |  |  |  |  |  |  |
| Dummy Lockdown | 0.0000171^***^ |  |  |  |  |  |
|  | (10.06) |  |  |  |  |  |
|  |  |  |  |  |  |  |
| Dummy Lockdown 10 days |  | 0.00000198 |  |  |  |  |
|  |  | (1.09) |  |  |  |  |
|  |  |  |  |  |  |  |
| Dummy Lockdown 20 days |  |  | -0.0000151^***^ |  |  |  |
|  |  |  | (-8.21) |  |  |  |
|  |  |  |  |  |  |  |
| Dummy Lockdown 25 days |  |  |  | -0.0000184^***^ |  |  |
|  |  |  |  | (-10.02) |  |  |
|  |  |  |  |  |  |  |
| Dummy Lockdown 30 days |  |  |  |  | -0.0000215^***^ |  |
|  |  |  |  |  | (-11.65) |  |
|  |  |  |  |  |  |  |
| Dummy Lockdown 40 days |  |  |  |  |  | -0.0000225^***^ |
|  |  |  |  |  |  | (-11.60) |
|  |  |  |  |  |  |  |
| Constant | 0.00000534^***^ | 0.0000100^***^ | 0.0000127^***^ | 0.0000127^***^ | 0.0000126^***^ | 0.0000120^***^ |
|  | (3.12) | (5.97) | (7.69) | (7.74) | (7.72) | (7.36) |
| Observations | 3380 | 3380 | 3380 | 3380 | 3380 | 3380 |
| Chi 2 | 464.7 | 356.3 | 428.6 | 464.6 | 503.1 | 502.1 |
| Rho | 0.0351 | 0.0335 | 0.0341 | 0.0345 | 0.0349 | 0.0346 |
| R2 | 0.149 | 0.154 | 0.186 | 0.195 | 0.204 | 0.202 |

*t* statistics in parentheses ^*^ *p* < 0.1, ^**^ *p* < 0.05, ^***^ *p* < 0.01

Table SM 5b - Random Effects - Worst quartile per Rule of Law

|  | (1) | (2) | (3) | (4) | (5) | (6) |
| --- | --- | --- | --- | --- | --- | --- |
|  | New Cases pc | New Cases pc | New Cases pc | New Cases pc | New Cases pc | New Cases pc |
| YCases pc | 0.0411^***^ | 0.0414^***^ | 0.0411^***^ | 0.0411^***^ | 0.0410^***^ | 0.0414^***^ |
|  | (57.33) | (57.31) | (56.80) | (56.62) | (56.47) | (57.45) |
|  |  |  |  |  |  |  |
| Dummy Lockdown | 0.000000592 |  |  |  |  |  |
|  | (1.54) |  |  |  |  |  |
|  |  |  |  |  |  |  |
| Dummy Lockdown 10 days |  | 6.60e-08 |  |  |  |  |
|  |  | (0.17) |  |  |  |  |
|  |  |  |  |  |  |  |
| Dummy Lockdown 20 days |  |  | 0.000000517 |  |  |  |
|  |  |  | (1.22) |  |  |  |
|  |  |  |  |  |  |  |
| Dummy Lockdown 25 days |  |  |  | 0.000000677 |  |  |
|  |  |  |  | (1.52) |  |  |
|  |  |  |  |  |  |  |
| Dummy Lockdown 30 days |  |  |  |  | 0.000000899^*^ |  |
|  |  |  |  |  | (1.90) |  |
|  |  |  |  |  |  |  |
| Dummy Lockdown 40 days |  |  |  |  |  | -7.58e-08 |
|  |  |  |  |  |  | (-0.14) |
|  |  |  |  |  |  |  |
| Constant | 0.000000209 | 0.000000432^*^ | 0.000000320 | 0.000000304 | 0.000000288 | 0.000000463^**^ |
|  | (0.84) | (1.86) | (1.46) | (1.42) | (1.38) | (2.31) |
| Observations | 3380 | 3380 | 3380 | 3380 | 3380 | 3380 |
| Chi 2 | 3550.7 | 3545.9 | 3548.9 | 3550.5 | 3553.2 | 3545.9 |
| Rho | 0 | 0 | 0 | 0 | 0 | 0 |
| R2 | 0.513 | 0.512 | 0.512 | 0.513 | 0.513 | 0.512 |

*t* statistics in parentheses ^*^ *p* < 0.1, ^**^ *p* < 0.05, ^***^ *p* < 0.01

Table SM 6a - Random Effects - Best quartile per Regulatory Quality

|  | (1) | (2) | (3) | (4) | (5) | (6) |
| --- | --- | --- | --- | --- | --- | --- |
|  | New Cases pc | New Cases pc | New Cases pc | New Cases pc | New Cases pc | New Cases pc |
| YCases pc | 0.00520^***^ | 0.00928^***^ | 0.0137^***^ | 0.0142^***^ | 0.0144^***^ | 0.0132^***^ |
|  | (7.59) | (12.88) | (19.61) | (20.97) | (22.13) | (21.99) |
|  |  |  |  |  |  |  |
| Dummy Lockdown | 0.0000179^***^ |  |  |  |  |  |
|  | (10.11) |  |  |  |  |  |
|  |  |  |  |  |  |  |
| Dummy Lockdown 10 days |  | 0.00000165 |  |  |  |  |
|  |  | (0.87) |  |  |  |  |
|  |  |  |  |  |  |  |
| Dummy Lockdown 20 days |  |  | -0.0000165^***^ |  |  |  |
|  |  |  | (-8.66) |  |  |  |
|  |  |  |  |  |  |  |
| Dummy Lockdown 25 days |  |  |  | -0.0000198^***^ |  |  |
|  |  |  |  | (-10.43) |  |  |
|  |  |  |  |  |  |  |
| Dummy Lockdown 30 days |  |  |  |  | -0.0000228^***^ |  |
|  |  |  |  |  | (-11.99) |  |
|  |  |  |  |  |  |  |
| Dummy Lockdown 40 days |  |  |  |  |  | -0.0000232^***^ |
|  |  |  |  |  |  | (-11.65) |
|  |  |  |  |  |  |  |
| Constant | 0.00000652^***^ | 0.0000111^***^ | 0.0000136^***^ | 0.0000135^***^ | 0.0000134^***^ | 0.0000127^***^ |
|  | (3.72) | (6.48) | (8.02) | (8.03) | (7.97) | (7.62) |
| Observations | 3380 | 3380 | 3380 | 3380 | 3380 | 3380 |
| Chi 2 | 446.9 | 337.3 | 418.0 | 454.7 | 492.5 | 484.1 |
| Rho | 0.0362 | 0.0346 | 0.0353 | 0.0357 | 0.0361 | 0.0358 |
| R2 | 0.138 | 0.145 | 0.179 | 0.188 | 0.196 | 0.193 |

*t* statistics in parentheses ^*^ *p* < 0.1, ^**^ *p* < 0.05, ^***^ *p* < 0.01

Table SM 6b - Random Effects - Worst quartile per Regulatory Quality

|  | (1) | (2) | (3) | (4) | (5) | (6) |
| --- | --- | --- | --- | --- | --- | --- |
|  | New Cases pc | New Cases pc | New Cases pc | New Cases pc | New Cases pc | New Cases pc |
| YCases pc | 0.0218^***^ | 0.0213^***^ | 0.0205^***^ | 0.0202^***^ | 0.0211^***^ | 0.0226^***^ |
|  | (15.02) | (14.40) | (13.51) | (13.13) | (13.43) | (14.08) |
|  |  |  |  |  |  |  |
| Dummy Lockdown | 0.00000332^***^ |  |  |  |  |  |
|  | (4.14) |  |  |  |  |  |
|  |  |  |  |  |  |  |
| Dummy Lockdown 10 days |  | 0.00000374^***^ |  |  |  |  |
|  |  | (4.42) |  |  |  |  |
|  |  |  |  |  |  |  |
| Dummy Lockdown 20 days |  |  | 0.00000467^***^ |  |  |  |
|  |  |  | (5.03) |  |  |  |
|  |  |  |  |  |  |  |
| Dummy Lockdown 25 days |  |  |  | 0.00000490^***^ |  |  |
|  |  |  |  | (4.94) |  |  |
|  |  |  |  |  |  |  |
| Dummy Lockdown 30 days |  |  |  |  | 0.00000366^***^ |  |
|  |  |  |  |  | (3.39) |  |
|  |  |  |  |  |  |  |
| Dummy Lockdown 40 days |  |  |  |  |  | 0.00000178 |
|  |  |  |  |  |  | (1.35) |
|  |  |  |  |  |  |  |
| Constant | 7.53e-08 | 0.000000220 | 0.000000316 | 0.000000457 | 0.000000827^*^ | 0.00000121^***^ |
|  | (0.13) | (0.41) | (0.62) | (0.91) | (1.68) | (2.58) |
| Observations | 3380 | 3380 | 3380 | 3380 | 3380 | 3380 |
| Chi 2 | 310.4 | 312.9 | 318.9 | 318.0 | 304.4 | 298.1 |
| Rho | 0.00356 | 0.00357 | 0.00359 | 0.00359 | 0.00354 | 0.00310 |
| R2 | 0.0954 | 0.0959 | 0.0972 | 0.0969 | 0.0937 | 0.0915 |

*t* statistics in parentheses ^*^ *p* < 0.1, ^**^ *p* < 0.05, ^***^ *p* < 0.01
